# SABCS 2021 Insights on Breast Cancer Research: An Interview with Dr. Laura Spring

**DOI:** 10.1093/oncolo/oyac008

**Published:** 2022-03-28

**Authors:** Anne Jacobson

## Abstract

Laura Spring, MD, a clinical investigator and breast medical oncologist at Massachusetts General Hospital Cancer Center and Harvard Medical School, shared insights on breast cancer research presented at the 2021 San Antonio Breast Cancer Symposium (SABCS).


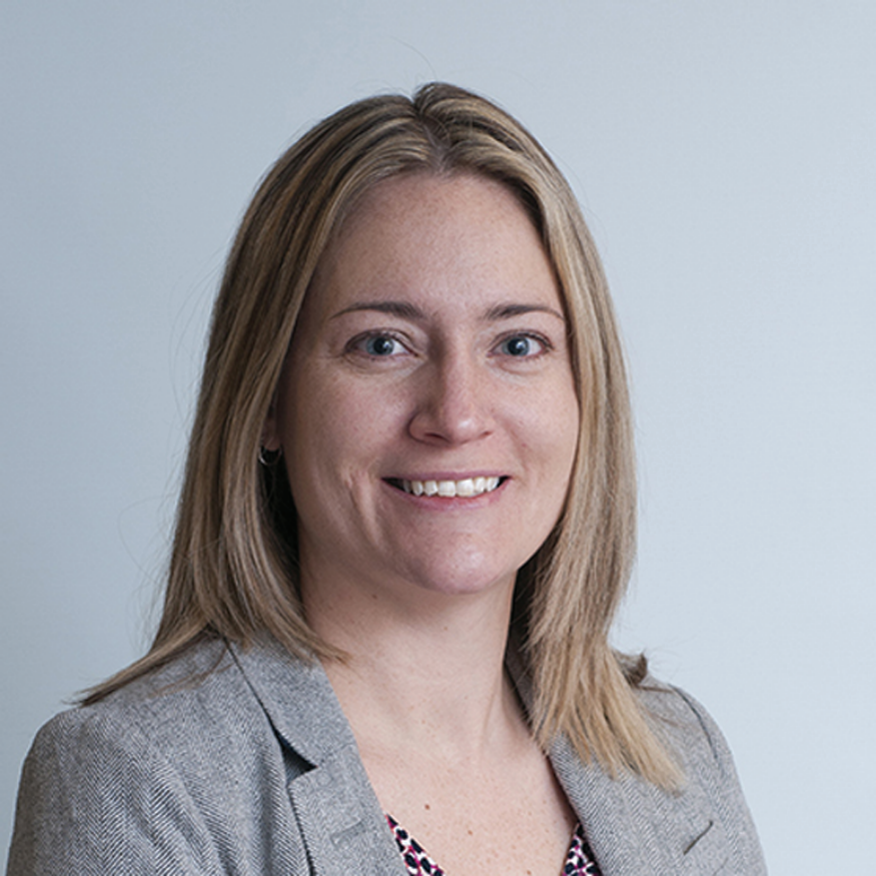


Laura Spring, MD

***The Oncologist:* Starting with human epidermal growth factor receptor 2 (HER2)-positive breast cancer, we saw updates from the DESTINY-Breast03 trial, which compared trastuzumab deruxtecan and trastuzumab emtansine in patients with HER2-positive metastatic breast cancer. What are your thoughts on this trial, particularly the findings in patients with brain metastases?**

**Dr. Spring:** The DESTINY-Breast03 subgroup results were great to see. In particular, the development of brain metastases is a major issue in HER2-positive metastatic breast cancer, and we need more therapeutic strategies. We had previously seen some data suggesting trastuzumab deruxtecan may have some intracranial activity. With this update from DESTINY-Breast03, now we see further results showing many patients with brain metastases did benefit from trastuzumab deruxtecan, further suggesting that this can be an option for patients with brain metastases as well.

***The Oncologist*: Another trial in the HER2-positive metastatic setting was the phase III PHOEBE trial comparing pyrotinib plus capecitabine with lapatinib plus capecitabine after prior treatment with trastuzumab and taxanes. What are the key take-home messages from this trial?**

**Dr. Spring:** Pyrotinib outperformed lapatinib, but the PHOEBE trial occurred during a time of active drug development in the HER2-positive space, making it difficult to know how the agent will fit into the landscape. However, being an oral agent, it may be a good option in areas where access to certain anti-HER2 therapies is limited.

***The Oncologist*: We also saw several trials in hormone receptor (HR)-positive, HER2-negative breast cancer. The phase III EMERALD trial evaluated elacestrant in patients with previously treated estrogen receptor (ER)-positive metastatic breast cancer. What are your thoughts on this trial?**

**Dr. Spring:** The EMERALD trial was the first phase III study of a novel oral selective estrogen receptor degrader (SERD) to demonstrate a statistically significant improvement in progression-free survival (PFS) versus standard of care. The results suggest elacestrant may become a new treatment option for patients with metastatic breast cancer. The results were more impressive in patients with *ESR1* mutations. Both arms of the study had a relatively short PFS, suggesting more work is needed to address the issue of endocrine-resistant disease.

Being first in class is always important and has potential to lead to an approval. Although elacestrant outperformed the treatment of physician’s choice (fulvestrant or an aromatase inhibitor), the outcomes overall were somewhat disappointing in either arm. This highlights that we still have a lot of work to do for this disease. Many of these patients were likely resistant to estrogen-blocking therapy—this is subset that needs a lot of research focus. Ultimately the novel oral SERDS may be even more helpful in the adjuvant breast cancer setting, and trials are ongoing and planned.

***The Oncologist*: The phase III PADA-1 trial examined the strategy of switching from palbociclib plus an aromatase inhibitor to palbociclib plus fulvestrant upon detection of the *ESR1* resistance mutation in patients with ER-positive metastatic breast cancer. What can we learn from this trial?**

**Dr. Spring:** PADA-1 was an interesting study that looked at the impact of *ESR1* mutations detected via circulating tumor DNA (ctDNA). These are mutations that typically breast ­cancers don’t have right off the bat; they develop later as a resistance mechanism to certain types of endocrine therapy. This trial asked, if you identify *ESR1* mutations earlier and change the endocrine therapy backbone from an aromatase inhibitor to fulvestrant, is that helpful? This was an interesting proof-of-principle study. I don’t think it will change the standard of care right away, but it will influence other studies and it will be important to explore this paradigm more.

***The Oncologist*: Updates from the phase III MONALEESA trials, which evaluated ribociclib plus endocrine therapy in patients with HR-positive, HER2-negative advanced breast cancer, focused on treatment outcomes across subgroups. What are the take-home messages from these updates?**

**Dr. Spring:** The updates from MONALEESA continued to show a treatment benefit with ribociclib regardless of tumor subtype or site of metastatic disease. Overall survival benefit was consistent across the intrinsic subtypes (luminal A, luminal B, and HER2-enriched), which is important because the intrinsic subtypes speak to disease biology. These different subtypes can behave quite differently, so it’s nice to see that ribociclib was still helpful regardless. In terms of the number and sites of metastases, which speaks to burden of disease, treatment with ribociclib was again helpful regardless of the situation.

***The Oncologist*: Several updates from the phase II BYLieve trial also examined outcomes across patient subgroups—in this case, patients with HR-positive, HER2-negative, *PIK3CA*-mutated advanced breast cancer who progressed on or after prior treatment with a cyclin dependent kinase (CDK) 4/6 inhibitor. What does this research tell us about the ability to identify predictive biomarkers?**

**Dr. Spring:** The BYLieve trial is important because the study that led to the approval of alpelisib, SOLAR-1, included relatively few patients treated with a CDK 4/6 inhibitor, as the standard of care was evolving at that time. The recent data presented at SABCS 2021 helps confirm the efficacy of alpelisib, even in patients with a short duration of prior treatment with CDK 4/6 inhibitors or with *ESR1* mutations. A short duration of response to CDK 4/6 inhibitors and the presence of an *ESR1* mutation can both suggest endocrine resistance, and so it was nice to see that alpelisib plus fulvestrant showed efficacy in these settings. Like many targeted therapies, alpelisib has side effects; ultimately it would be great for patients if we could better predict who may benefit more, and who may benefit less, from a drug. There is also interest in biomarkers that may help predict side effects, to help with personalization. As we heard with the EMERALD study, we’re going to continue to have more agents available, and therefore personalization of therapy selection becomes even more important.

***The Oncologist*: Yes, speaking of personalized treatment, we also saw an update from the phase III Southwest Oncology Group (SWOG) S1007 RxPonder trial, which evaluated the benefit of adjuvant chemotherapy based on clinical and genetic risk factors in patients with HR-positive, HER2-negative, early breast cancer. What have we learned about personalizing therapy in this setting?**

**Dr. Spring:** This update confirmed previous overall findings from the RxPonder trial, which has helped many patients avoid unnecessary treatment with chemotherapy. One challenge involves the premenopausal subset, which continues to show benefit from adjuvant therapy regardless of Oncotype DX recurrence score according to the study results. The most recent RxPonder update reviewed how many premenopausal patients received ovarian function suppression (OFS), and that number was relatively low (6.3% in the chemoendocrine arm and 19.0% in the endocrine-only arm). Therefore, it is still an unanswered question whether OFS could replace chemotherapy and provide the same benefit. The RxPonder trial just wasn’t the right trial to answer the question, but it was informative to see data showing that not many premenopausal patients received OFS.

***The Oncologist*: Lastly, updates from the KEYNOTE-355 and KEYNOTE-522 trials examined the role of checkpoint inhibition with pembrolizumab in patients with triple-negative breast cancer (TNBC). What are your thoughts on these trials?**

**Dr. Spring:** Updated results from KEYNOTE-522 and KEYNOTE-355 continue to establish the role of pembrolizumab both in programmed death-ligand 1 (PDL-1)-positive metastatic TNBC and in stage II and III TNBC regardless of PDL-1 subtype. KEYNOTE-355 more specifically examined the PDL-1 cutoff that identifies patients who are most likely to benefit from pembrolizumab, whereas KEYNOTE-522 focused on the updated event-free survival data. The further follow-up data from both trials helped solidify the role of this agent becoming a standard of care in TNBC.

## Notes

Please note that this content was created for the purpose of summarizing the content of the San Antonio Breast Cancer Symposium 2021 and has not been peer reviewed under the standard policies of *The Oncologist*.

